# Diagnosis, surgery, and outcome of tethered cord syndrome in 12 dogs

**DOI:** 10.1093/jvimsj/aalaf031

**Published:** 2026-01-21

**Authors:** Rachel Lampe, Elina Kalamkarova, Laura Barnard, Augustin Mareschal, Erin K Keenihan, Nick J H Sharp

**Affiliations:** Department of Neurology, Canada West Veterinary Specialists, 1988 Kootenay Street, Vancouver, BC V5M4Y3S, Canada; Department of Neurology, Canada West Veterinary Specialists, 1988 Kootenay Street, Vancouver, BC V5M4Y3S, Canada; Department of Neurology, Canada West Veterinary Specialists, 1988 Kootenay Street, Vancouver, BC V5M4Y3S, Canada; Department of Radiology, Canada West Veterinary Specialists, 1988 Kootenay Street, Vancouver, BC V5M4Y3S, Canada; Department of Radiology, Canada West Veterinary Specialists, 1988 Kootenay Street, Vancouver, BC V5M4Y3S, Canada; Department of Neurology, Canada West Veterinary Specialists, 1988 Kootenay Street, Vancouver, BC V5M4Y3S, Canada

**Keywords:** tethered cord syndrome, neurosurgery, congenital, TCS

## Abstract

**Background:**

Tethered cord syndrome (TCS) results from tension on the conus medullaris (CM), causing pain, bladder or bowel dysfunction, and lower limb neurologic deficits in humans. It is underrecognized in dogs; diagnosis is difficult and depends on improvement after surgery.

**Hypothesis/Objectives:**

Describe clinical signs, advanced imaging results, surgical procedures, and outcomes of dogs with TCS. Develop objective measurements of CM and dural sac (DS) movement on dynamic magnetic resonance imaging (MRI) in dogs that benefitted from TCS surgery.

**Animals:**

Twelve client-owned dogs that underwent TCS surgery with follow-up.

**Methods:**

Retrospective study. Diagnosis was based on clinical signs and MRI or computed tomography (CT). The MRI measurements were performed retrospectively in extension and flexion.

**Results:**

Median age at presentation was 52 months (range, 1-12 years). All the dogs had lumbar pain and transient paresthesias such as biting at their hindquarters, sitting urgently or looking at their hindquarters. Dynamic imaging identified minimal craniocaudal movement of the DS and CM between flexion and extension. The mean movement of the CM and DS was 0 mm (range, −1.79 to −2.6 mm), and 0.93 mm (range, −1.4 to −2.9 mm), respectively. All the dogs had a taut external or internal filum terminale, extradural adhesions, or both, which were transected during surgery. All the dogs showed improvement at short-term and long-term follow-up.

**Conclusions and clinical importance:**

Dogs with unexplained lumbar pain and transient paresthesias should be evaluated using dynamic MRI. If minimal movement of the DS or CM is noted, surgical detethering is indicated.

## Introduction

**Figure 1:**
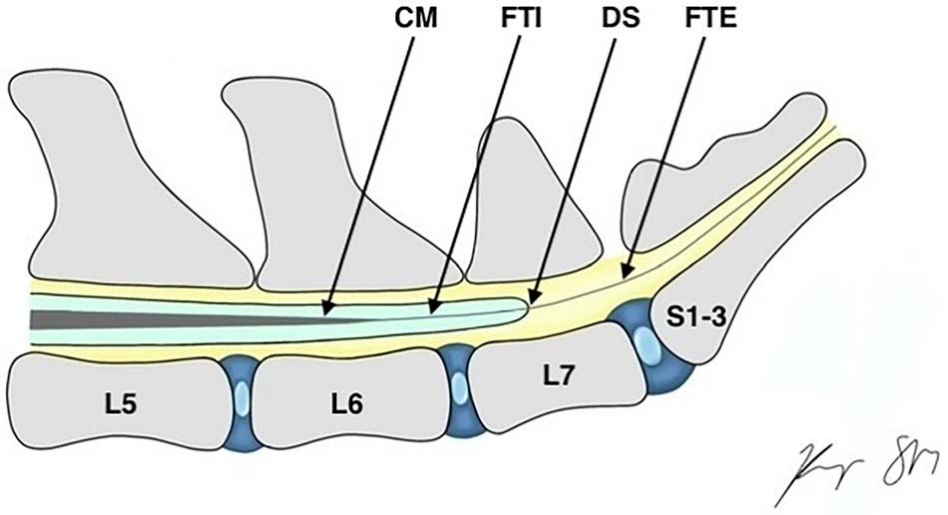
Anatomy of the CM and FT, showing internal and external filum connecting the CM to the paravertebral structures. Abbreviations: CM = conus medullaris; FTI = filum terminale internum; DS: dural sac; FTE = filum terminale externum.

In domestic mammals, neural tissue in the vertebral canal tapers to a point known as the conus medullaris (CM).^1^ The filum terminale (FT) connects the CM to the caudal vertebra.^1–4^ The FT is composed of two segments, the intradural filum terminale internum (FTI) and the extradural filum terminale externum (FTE). The FTI is surrounded by a dural sac (DS) until these structures fuse together to make the FTE, an extradural structure anchoring the CM to the vertebrae (Figure 1).^5^

Tethered cord syndrome (TCS) has been recognized in humans as either a mechanical tethering or an inelastic FT causing abnormal traction on the CM. It can be associated with neural tube defects (NTDs) or can occur as a result of FT abnormalities.^2^ It has been recognized in both pediatric and adult populations.^6–8^ Typical symptoms include at least two from the triad of back or leg pain, neurologic deficits including gait changes, and bladder dysfunction.^2^^,^^9^ Diagnosis relies on history, clinical signs, and magnetic resonance imaging (MRI) findings. Supportive MRI findings include an abnormally caudal CM known as a “low lying conus” or a thickened or fat-infiltrated FT.^2^^,^^9^ Filum terminale transection, detethering of adhesions, or both have been established as the treatment, often leading to clinical improvement after surgical intervention.^8^^,^^10^^,^^11^

Tethered cord syndrome without concurrent NTDs has been rarely reported in veterinary literature.^12–17^ One contemporaneous case series described TCS in 30 dogs, but only 11 had the diagnosis confirmed at surgery.^15^ These dogs presented with a combination of lumbosacral pain, hind limb weakness, behavior changes, bladder dysfunction, or some combination of these findings. The inclusion criteria for diagnosis were a L4-S3 myelopathy or cauda equina neurolocalization and normal MRI findings, and only 12 of 19 medically managed cases had dynamic MRI performed. Tethered cord syndrome is particularly challenging to diagnose in dogs because the CM location varies with both the size and breed of the dog.^4^^,^^18^

Our first objective was to retrospectively describe the clinical signs, imaging findings, surgical approach, and outcomes in a cohort of dogs with TCS. Our second objective was to quantify the extent of cranial-caudal movement of the CM and DS using dynamic imaging to provide an objective means of TCS diagnosis.

## Materials and methods

### Case selection

Medical records and imaging findings of dogs with a diagnosis of TCS that had surgery between May 2021 and October 2024 were reviewed retrospectively. All the dogs were diagnosed and treated at Canada West Veterinary Specialists. The inclusion criteria consisted of complete medical records, including signalment at diagnosis, presenting clinical signs and their duration, neurologic examination findings, and short-term postoperative follow-up. All the dogs were diagnosed using dynamic spinal computed tomography (CT) or MRI, and reviewed by a board-certified radiologist, confirming minimal movement of the DS, CM, or both from flexion to extension. All of the affected dogs had surgical detethering performed.

### Computed tomography or magnetic resonance imaging examination

Advanced imaging was performed with the dog in dorsal recumbency using either a 0.25 T MRI system (Esaote Vet-MR Grande), a 1.5 T Siemens Avanto MRI system, or a 64-slice CT unit (Toshiba Aquilion 64, Canon Medical Systems). Dynamic imaging was defined as imaging performed with the pelvic limbs in both flexed and extended positions. For extended positioning, the pelvic limbs were pulled caudally and secured with extension of the coxofemoral joints, and for flexed positioning the pelvic limbs were pulled cranially with extended stifles and maximally flexed coxofemoral joints.^19^ Minimum MRI sequences included sagittal T2W, T1W, and half-Fourier acquisition single-short turbo spin-echo (HASTE) images and transverse T2W images transecting the CM and DS. Additional sequences in selected dogs included transverse T2W sampling perfection with application-optimized contrast using different flip angle evolution (SPACE, a volumetric sequence), sagittal or dorsal T2W short τ inversion recovery (STIR), transverse T1W, fat-saturated sagittal (T1W or T2W), and postcontrast sagittal and transverse T1W images.

### Surgery

All the dogs underwent a lumbosacral dorsal laminectomy. Surgical procedures were performed on the dogs under general anesthesia, and they received perioperative pain management with a full μ-opioid and ketamine. Surgeries were performed by one of two board-certified neurologists (L.B. or R.L.). A dorsal laminectomy was performed centered over the termination of the DS. The vertebral canal was examined for abnormal extradural structures tethering the DS to the periosteum, and these structures were transected if identified. The FTE was identified and evaluated subjectively for tension. A durotomy also was performed to expose the FTI in some dogs, and the dural incision was extended caudally to the FTE. If there appeared to be excessive tension visible in the FTE or FTI, or both, they were transected. The specific surgical technique, or combination of techniques, used to relieve tension on the DS and CM was decided by the surgeon intraoperatively. Transected structures were submitted for histopathology when possible.

### Outcomes

All the dogs had short-term outcomes assessed by the overseeing neurologist at an in-person evaluation or phone call 4-12 weeks after surgery. Long-term outcomes, defined as >6 months after surgery, were assessed using a standardized survey sent by email (Supplementary Material S1). The survey asked owners to determine the degree of postoperative improvement (none, partial, or complete) in each of the identified common presenting clinical signs.

### Retrospective imaging analysis

After selection of cases to include, all the dynamic MRI studies were retrospectively examined and objective measurements of CM and DS movements were performed by a board-certified neurologist (R.L.). The CT scans were not analyzed for this portion of the study because the CM is not visible using this modality, and no normal data on CT imaging is available for comparison.

**Figure 2:**
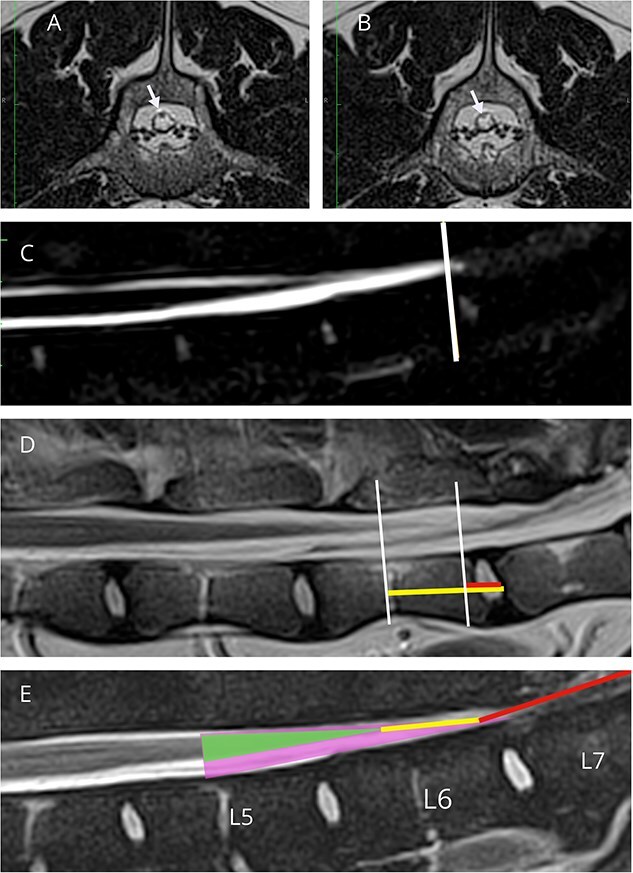
MRI images in extended position used for measurement of the caudal-most aspect of the CM and DS. (A) T2-weighted transverse image showing hypointense CM (white arrow) within cerebrospinal fluid (CSF) of the DS. (B) T2-weighted transverse slice just caudal to the level in (A) displaying loss of hypointense signal of the CM (white arrow). The slice in (A) corresponds to the cranial white line in (D) representing the most caudal aspect of the CM. (C) HASTE sequence with line showing the caudal-most aspect of the DS, at the caudal aspect of the brightest CSF signal, before it begins to taper off in intensity.^18^ In some dogs like the one shown here, the exact termination had to be estimated by assessing both the HASTE and transverse T2 images. (D) T2-weighted sagittal image with cranial and caudal lines representing the termination sites of the CM and DS respectively. Note that locations were confirmed using corresponding transverse images. Horizontal lines are drawn from the cranial endplate of L7 to the caudal-most aspect of the CM (horizontal longer yellow line) and DS (horizontal shorter red line) used for measurement. A negative distance indicated that the location was cranial to the L7 cranial endplate; conversely, a positive value indicated that it was caudal to the L7 cranial endplate. (E) T2W FatSat sagittal image annotated to show the anatomy of the cauda equina. The smaller (green) triangle represents the end of the CM, and the larger (purple) triangle represents the outline of the DS. The line inside the DS (yellow) represents the FTI, which then continues into the longer line (red), representing the FTE. Note: The FT is often not fully visible on MRI.

The lumbosacral angle was calculated to ensure appropriate dynamic imaging was performed in each dog, as described previously.^19^ Craniocaudal movement of the CM and DS was measured by identifying the end of the CM and DS in the flexed and extended views and measuring their distance from the cranial endplate of L7, using landmarks and methods described previously.^20^ The most caudal aspect of the CM was identified where the tapering CM transitioned to a structure of constant diameter or could no longer be identified. The CM is hypointense on T2W images as it is tapering and then becomes more isointense compared to the surrounding hyperintense cerebrospinal fluid. Therefore, the termination site of the CM was defined as the most caudal hypointense point in the DS, where it stopped tapering and before its signal intensity increased noticeably (Figure 2A and B). This finding was most evident using a T2W sagittal sequence and T2W or T2W SPACE transverse images.^20^^,^^21^ The dural sac termination was identified using the most caudal point of the brightest cerebrospinal fluid signal on T2W HASTE and T2W sagittal images, combined with T2W transverse images (Figure 2C), as described previously.^20^^,^^21^ The location of the DS was evaluated in flexed and extended positions, and identified as dorsally displaced if it was located dorsally against the vertebral lamina.

To measure the movement of the CM and DS with dynamic imaging, a line through the most caudal aspect of each location was drawn perpendicular to the long axis of the vertebral canal and intersecting with the vertebral body. The distance between this line and the center of the cranial endplate of L7 was recorded successively with the hindlimbs extended and flexed (Figure 2D).^20^ A negative distance indicated that the location was cranial to the L7 cranial endplate. Displacement was calculated by subtracting the distances measured in extended and flexed views.^20^ The vertebral level of the termination of the CM and DS also was recorded for each case. If the termination site was over a disc space, the more cranial vertebra was recorded (ex: if it terminated over the L6-7 intervertebral disc, then the location was recorded as L6).^18^ To standardize the CM and DS translocation with body size, the displacement for both the CM and DS was divided by the length of the dog’s L7 vertebral body length.

## Results

### Animals

Twelve dogs were diagnosed with TCS, underwent untethering surgery, and had short-term follow-up. Their median age at presentation was 52 months (range, 1-12 years), and the median weight was 13 kg (range, 9.6-35 kg). Nine dogs were male, and three were female. There were two Australian Shepherds, two Husky cross dogs, and one each of a Welsh Corgi, Labrador Retriever, Golden Retriever, Boston Terrier, Lakeland Terrier, Formosan Mountain Dog, and a German Shepherd. The median age of onset of clinical signs was 24 months (range, 2-126 months) or 2-47 months, excluding the 12-year-old dog. All the signs were reported to be initially subtle in onset and progressive in nature. The median duration of signs before presentation was 36 months (range, 6-49 months).

### Clinical findings and history

**Figure 3:**
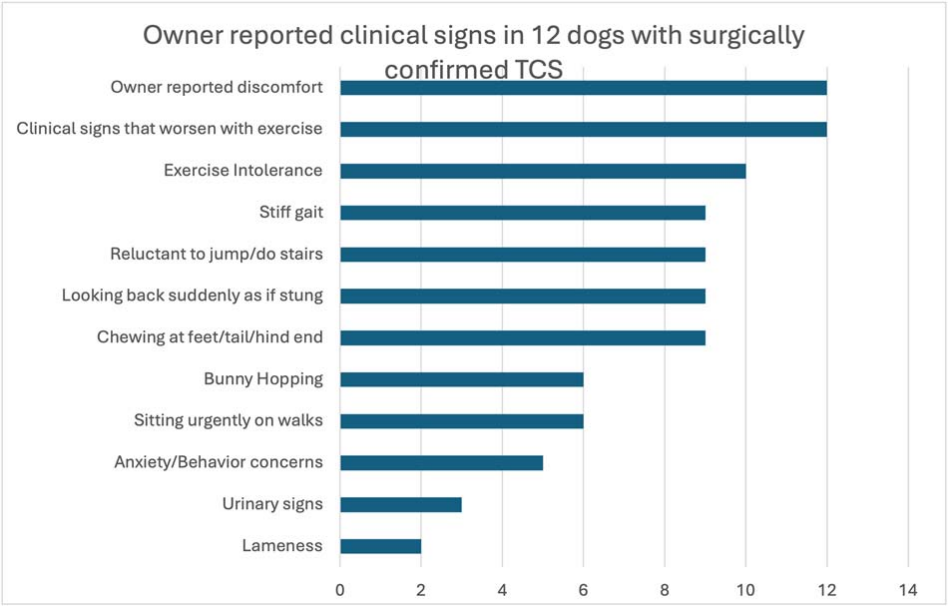
Reported clinical signs in the population of 12 dogs diagnosed with tethered cord syndrome that underwent surgery.

All the owners reported that their dogs’ clinical signs worsened with exercise, and reported an activity suggestive of transient paresthesia, such as looking suddenly at their hindquarters, chewing at the paw or tail, or sitting urgently while on walks. Eleven of the twelve dogs were reported to be uncomfortable, based on signs such as hypersensitivity to touch, grunting or yelping. The remaining clinical signs at presentation are listed in Figure 3. Notably, 5 of the 12 dogs had a history of excessive anxiety or presumed behavior disorders that could not be managed using anxiolytic medications. Three dogs had urinary incontinence and two dogs seemed uncomfortable while posturing to defecate (Supplementary Material S2). Three dogs had undergone previous MRI of the thoracolumbar vertebral column for the same complaint but TCS had not been recognized.

All the dogs had unremarkable physical examinations, including strong and synchronous femoral pulses. Neurologic examination findings were similar in most dogs, including a normal or stiff pelvic limb gait. All the dogs had either pain on lumbar palpation (*n* = 10) or pain while extending their hips (*n* = 6). Pain worsened when the lumbar vertebral column was palpated with the hips elevated. Three dogs had delayed paw-placing reactions, weak withdrawals, or both in the hind limbs (Supplemental Material S2). One dog had mild ataxia and ambulatory paraparesis, with delayed paw-placing reactions in the hind limbs and was initially localized as a T3-L3 myelopathy, but imaging of the thoracolumbar vertebral column indicated no abnormalities to explain those signs. The remaining neurologic examinations were normal. Neurologic localization based on neurologic examination was consistent with an L4-S3 or cauda equina (5), or solely lumbar pain (6) in all the dogs apart from the one previously described.

All the dogs presented to our clinic after trials of pain relief medications, including combinations of gabapentinoids, methocarbamol, anxiolytics, prednisone, and nonsteroidal anti-inflammatory drugs, with either no or mild improvement. All the dogs had seen other specialists or rehabilitation therapists, or both, for their concerns. After unsuccessful medical management, each owner elected to proceed with diagnostic imaging.

### Diagnostic testing, treatment, and outcomes

#### Diagnosis

**Figure 4:**
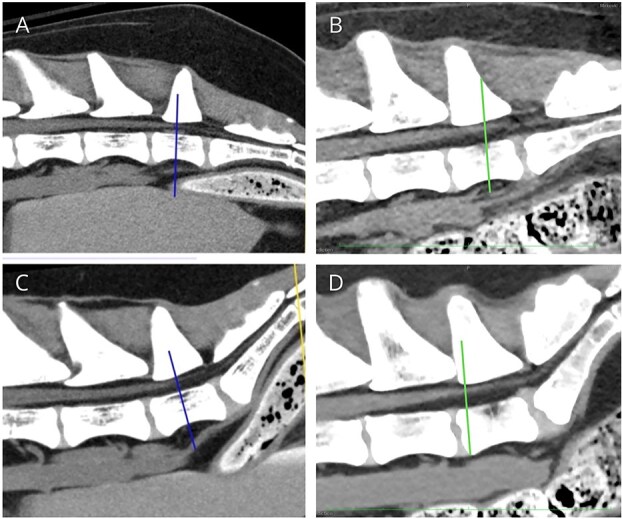
Dynamic CT of a dog with TCS (A and C), and a normal dog (B and D). (A) and (B) are in a flexed position, whereas (C) and (D) are in an extended position. Lines are drawn at the end of the DS/start of the FTE. The CT was viewed in multiplanar reconstruction, which allowed the use of transverse images to identify the termination of the DS at the location it stopped tapering; then the corresponding line was drawn on the sagittal images. The CM is not visible on CT imaging. In (A) and (C), the DS ends at mid L7, with limited movement between the two views. Panels (B) and (D) show translocation of the DS cranially with extension, with the DS moving almost half a vertebral length.

**Figure 5:**
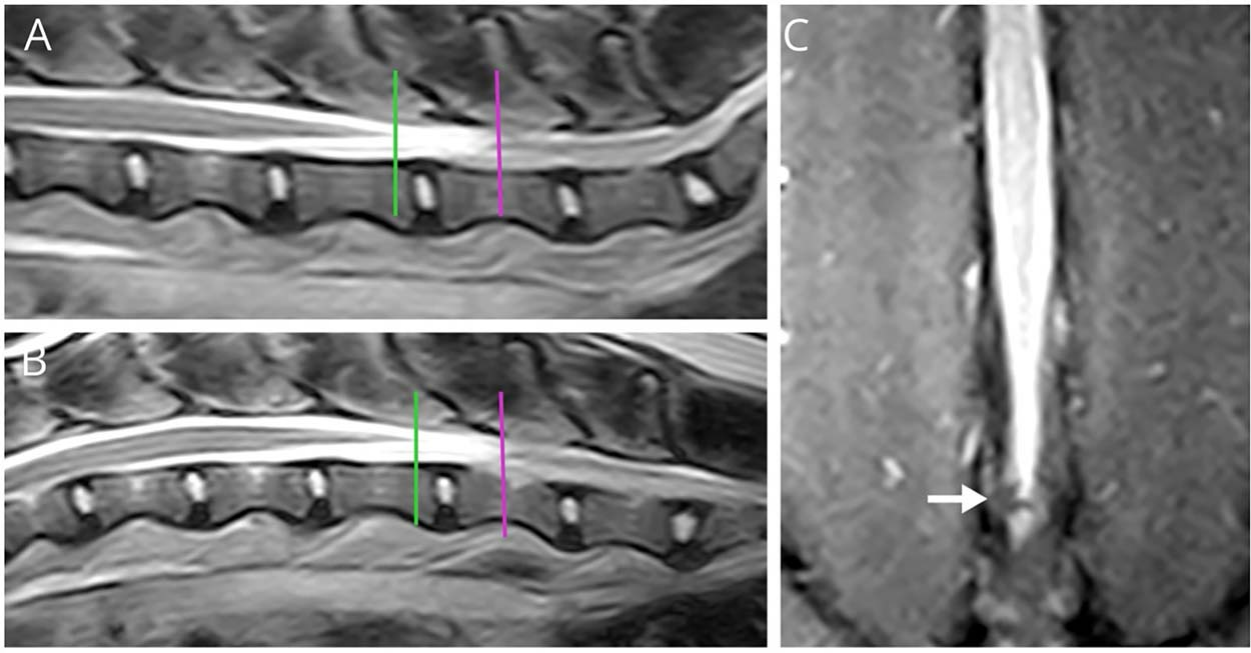
Dynamic MRI of the lumbar vertebral column in a dog with tethered cord syndrome, showing a lack of movement of the CM (cranial line) and DS (caudal line) between extension (A) and flexion (B). (A) T2W sagittal of the lumbar vertebral column with legs positioned in extension. (B) T2W sagittal of the lumbar vertebral column with legs positioned in flexion. (C) Dorsal STIR sequence of the lumbar vertebral column, with the arrow pointing at an extradural lipomatous structure that was adhered to the spinal cord and was contributing to tethering.

All the dogs had normal CBCs and serum biochemistry panels. Two dogs had normal thoracic radiographs (including the 12-year-old dog). Diagnosis of TCS was made by dynamic MRI in 9 dogs and by dynamic CT in 3 dogs. Two of the dogs diagnosed by CT also had a nondynamic MRI of the lumbosacral region. The CT diagnosis was made based on lack of movement of the DS between flexion and extension (Figure 4), and MRI diagnosis was made based on a lack of movement of both the CM and DS (Figure 5). One dog had 8 lumbar vertebrae and an incompletely fused sacrum, and another had a sacral osteochondrosis dissecans lesion that was not compressive. One dog had a small extradural structure located within the right side of the vertebral canal at the level of L7 that was hyperintense on T2W images and suppressed on T2W STIR (Figure 5), consistent with a lipoma impinging on the DS. No other imaging abnormalities were reported, and if visualized, the FT was normal in appearance. Cerebrospinal fluid was collected from the lumbar cistern in 3 dogs and was normal on cytologic analysis.

#### Retrospective imaging analysis

**Figure 6:**
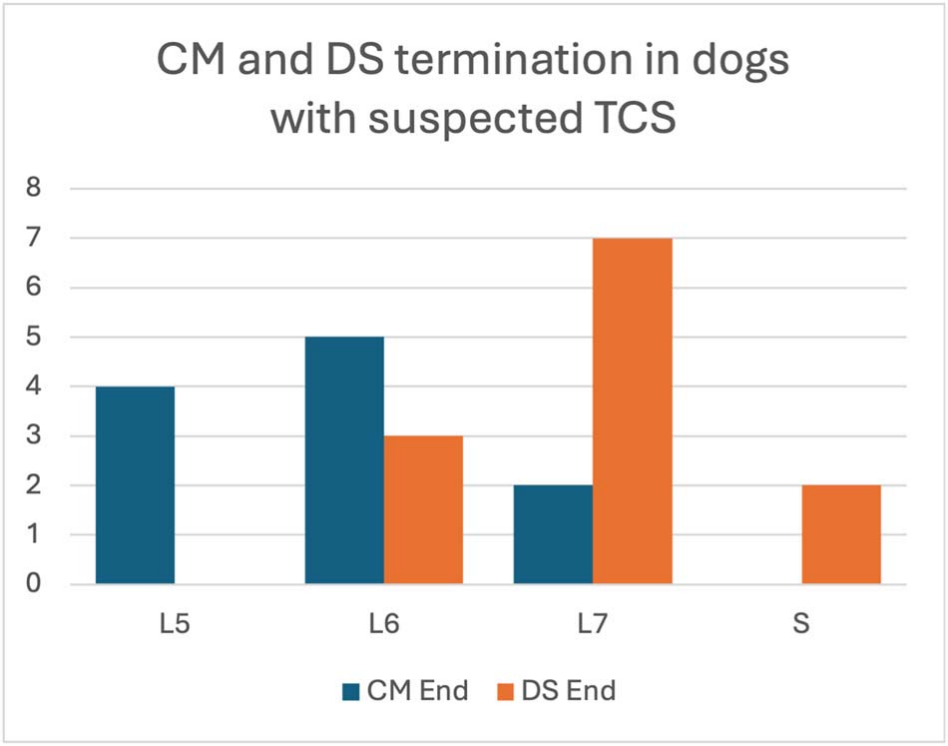
Conus medullaris (CM) and dural sac (DS) termination points on MRI and CT based on the associated vertebral location. If only CT was available, then only the DS termination site was recorded. No clinically relevant movement was noted between flexed and extended views, so termination points were only record in one location.

Measurements of CM and DS translocation were performed in the 9 dogs using dynamic MRI studies. The average lumbosacral angles in the flexed and extended position were 173 degrees (range, 170-177) and 149 degrees (range, 139-162), respectively, with an average angle change of 24 degrees (range, 12-34) from flexed to extended positions. The mean ± SD movement of the CM from extended to flexed position was 0.97 ± 1.32 mm, and the mean DS movement from extended to flexed positions was 1.24 ± 1.29 mm (Table 1). The CM was found to end between the fifth and seventh lumbar vertebral levels in all the dogs, with L6 being the most common termination site. The DS ended between the level of the sixth lumbar vertebrae and the sacrum, with L7 being the most common termination site (Figure 6). The DS was dorsally located in both flexed and extended positions in 5 dogs, and in 4 dogs was not against the dorsal lamina in either flexed or extended positions.

**Table 1:** Movement of the CM and DS with dynamic MRI, in dogs with TCS.^a^

	**Movement from extension to flexion**	
	**Mean, mm +/− SD**	**Range, mm**
**Conus medullaris**	0.97 +/− 1.3	−1.79 to 2.6
**Dural sac**	1.24 +/− 1.29	−1.4 to 2.9
**CM:L7**	−0.00 +/− 0.07	0 to 0.11
**DS:L7**	0.05 +/− 0.08	0 to 0.16

^a^Average movement of the CM and DS in 9 dogs with dynamic MRI studies of the lumbar vertebral column, in flexed and extended positions. When evaluating translocation of the CM and DS, a negative value indicates cranial displacement, while a positive value indicates caudal displacement (from extension to flexion). The CM:L7 and DS:L7 ratios were calculated by dividing the translocation of the CM and DS by the length of L7, to normalize for variation in dog size.

### Surgery

**Figure 7:**
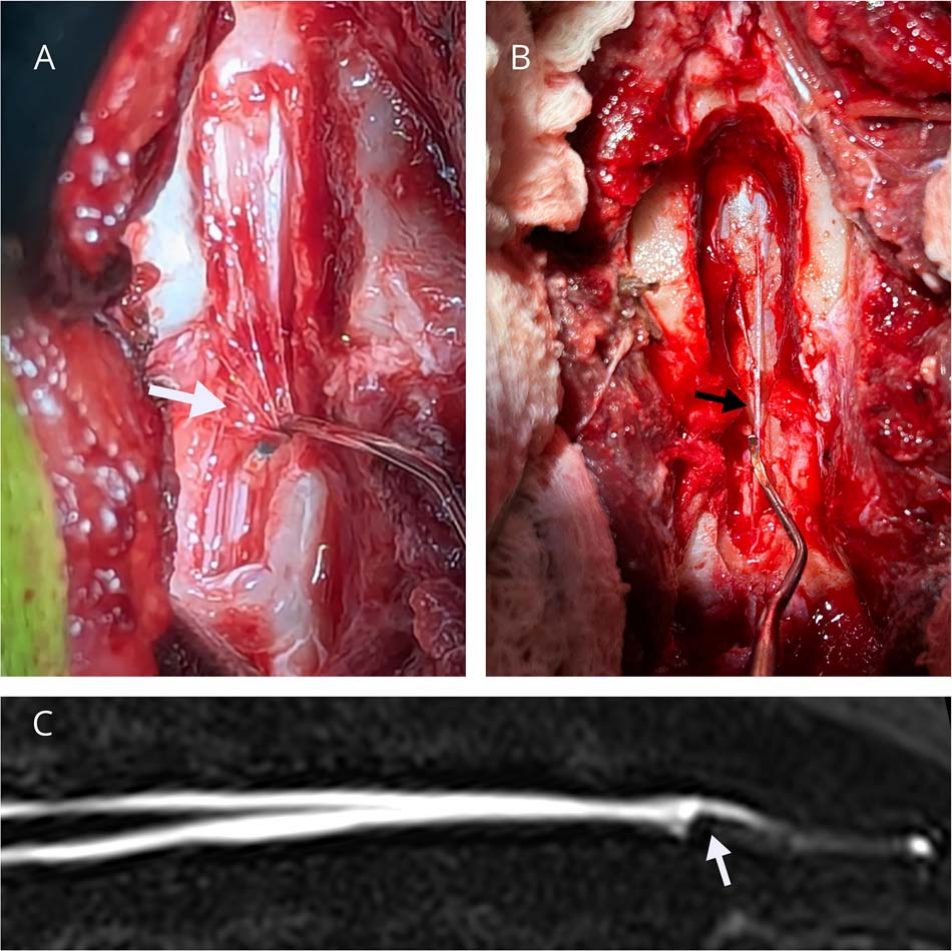
(A and B) Dorsal intraoperative view of the L7-S1 dorsal laminectomy in two dogs, showing extradural adhesions to the dural sac (arrows). A nerve root retractor is putting caudal traction on extradural adhesions leading to tension and caudal movement on the DS. (C) HASTE image of the lumbosacral vertebral column from the dog in (A), with the white arrow pointing at distortion of the CSF signal at the caudal aspect of the dural sac, suspected to be secondary to extradural tethering.

All the dogs underwent lumbosacral dorsal laminectomy over L7 and S1. In 4 dogs, the laminectomy extended into L6, and in 4 dogs it extended into S2. The laminectomy window was chosen to allow adequate visualization of the termination of the DS, based on imaging. All the dogs had at least one subjectively taut structure within the vertebral canal causing traction and increased tension on the DS. In 5 dogs, only the FTE appeared under tension. In another 5 dogs, in addition to a taut FTE, there were taut extradural, fibrofilamentous structures that appeared to be anchoring the DS to a sacral or caudal vertebra dorsally (Figure 7 and Supplementary Video S1). In one dog, the tension was only apparent on an extradural structure connected to the DS, whereas the FTE did not appear under tension. In the final dog, there was a taut FTE, as well as a discrete lipomatous structure attached to the exterior of the dura, which was removed by gentle traction and submitted for histopathology. In summary, 5 of the 12 dogs had tension visible only on the FTE, whereas 7 of the dogs had evidence of extradural tethering. The DS also appeared subjectively distended in 8 dogs. Durotomy was performed in 11 dogs. The FTE was transected in 9 dogs, and the FTI was transected in 2 dogs; one of these dogs had both the FTE and FTI transected. In 2 dogs, only fibrous extradural structures were transected, without transection of either FT. In one of these dogs, both the extradural adhesions and FTE initially appeared under tension. However, after the taut extradural adhesions were transected, the FTE and DS no longer appeared under tension, and thus the FT was not transected.

Dogs were hospitalized after surgery for 1-2 days and received IV analgesic drugs, including a full μ-opioid with or without ketamine for 12-24 hours. Orally administered analgesics, including an anti-inflammatory drug and a gabapentinoid, were started postoperatively and continued at home. Discharge instructions included 1 month of strict rest with only short (10-minute) walks several times a day, followed by a gradual increase in activity over the next 2 months. If dogs were doing well, full activity was allowed by 3 months postoperatively.

The histopathology of the various transected structures from 10 dogs consisted of unremarkable fibrous tissue, with variable amounts of adipose and hemorrhagic tissue intermixed. One of the two FTI samples also contained nerve fibers, whereas none of the FTE samples contained nerve fibers. The isolated lipomatous extradural structure from one dog was predominantly adipose-rich stroma abutting dense fibrous stroma with some focal fibrous stroma and rare epithelioid cells, but this structure did not resemble a typical FT. This structure was suspected to be abnormal epidural fat attached to the DS, contributing to tethering. No evidence of inflammatory or neoplastic cells was found in any sample.

No major perioperative complications were encountered, and all the dogs recovered uneventfully from anesthesia. Six dogs had transient, postoperative neurologic deterioration characterized by decreased voluntary tail movement and tone. One of these dogs also had absent tail sensation the day after surgery, and recovered 12 hours later (this dog had FTI transection). All the dogs had normal tail function at short-term follow-up. Two dogs developed new transient paw-placing deficits postoperatively in the pelvic limbs that had not been present before surgery, but these resolved entirely within a month. All the dogs remained ambulatory postoperatively, and none experienced new signs of urinary or fecal incontinence.

### Follow-up

Short-term follow-up between 4 and 12 weeks after surgery showed improvement in all the dogs. Back pain was reported by owners to be improved in 4 dogs and resolved in 8 dogs. Six of the nine dogs that had licked or chewed at their feet or hindquarters before surgery stopped this behavior completely. Of the three with persistent licking or chewing behavior, one would sometimes bite at the base of its tail less frequently than before surgery, one would lick at its inner thighs, again less aggressively than before surgery, and one occasionally licked its paws. Four owners reported that their dogs could sit straighter or with less difficulty or in postures not previously observed, such as like a sphinx or upright on their haunches. At short-term follow-up, 7/12 dogs were no longer receiving pain medications. Of the 5 dogs still on PO medications, 2 were still receiving prednisone at a lower dose than before surgery (0.15 and 0.25 mg/kg/day), 3 dogs were still on gabapentin (but one owner reported it was used for chronic anxiety rather than pain), and 1 dog was still on pregabalin. The dog that had shown ataxia and paresis before surgery continued to show these gait abnormalities afterward.

Long-term follow-up *>* 6 months after surgery was available in 11/12 cases. One dog was lost to follow-up. Eleven owners responded to the survey about the improvement of signs of TCS at a mean of 13 months (range, 6-28 months) after surgery (Table 2). In 7 of these 11 dogs, the owners reported partial or complete resolution of all signs present before surgery. All the owners reported substantially improved back pain, and 8/9 dogs reported improvement in reluctance to jump or climb stairs. All owners also reported substantial improvement in their dogs’ signs grouped as transient paresthesia, specifically chewing at the paws or hindquarters, looking suddenly at their hindquarters, and sitting urgently while on walks. Improvement was least likely to be reported in the gait category (lameness, bunny-hopping, and stiff gait), with 5/11 dogs having gait changes that did not improve after surgery. More specifically, only 57% of dogs that bunny-hopped experienced any improvement in this clinical sign. In the free comment section of the survey, 6 owners provided additional subjective comments that their dogs appeared to be more playful, less reactive, and easier to handle. Despite only partial resolution of many signs, 100% of the owners reported their dogs had an improved quality of life after surgery.

**Table 2:** Survey results of long-term changes in common TCS symptoms following surgery, as perceived by owners.^a^

	**No change**	**Partial resolution**	**Complete resolution**	**Total dogs**
**Pain**				
** Back pain, *n* (%)**	0	7 (64)	4 (36)	11
** Reluctance to jump/do stairs, *n* (%)**	1 (11)	6 (67)	2 (22)	9
**Gait, *n* (%)**				
** Stiff gait, *n* (%)**	2 (22)	5 (56)	2 (22)	10
** Bunny-hopping gait, *n* (%)**	3 (43)	3 (43)	1 (14)	7
** Lameness, *n* (%)**	1 (17)	1 (17)	4 (67)	6
**Transient paresthesias**				
** Looking back urgently as if stung, *n* (%)**	0	4 (44)	5 (56)	9
** Sitting urgently on walks, *n* (%)**	0	5 (56)	4 (44)	9
** Chewing at feet/hind end, *n* (%)**	0	6 (75)	2 (25)	8
**Other**				
** Exercise intolerance, *n* (%)**	2 (22)	5 (56)	2 (22)	9
** Signs that worsen with exercise, *n* (%)**	1 (10)	8 (80)	1 (10)	10
** Anxiety/behavior changes, *n* (%)**	1 (14)	4 (57)	2 (29)	7
** Urinary incontinence, *n* (%)**	1 (33)	0	2 (66)	3

^a^Survey results of owner perception of changes in common tethered cord signs at long-term follow-up after tethered cord surgery. Surveys were completed an average of 13 months after surgery (range 6-28 months). Results are based on owner’s observations, and it was interesting to note there were some discrepancies between owner’s answers on survey and those provided in person (Supplementary Material S4).

## Discussion

In our retrospective study, we describe 12 dogs with TCS that underwent surgical detethering, all of which showed improvement in clinical signs after surgery. Tethered cord syndrome is likely underdiagnosed in dogs because clinical signs are nonspecific and standardized imaging criteria do not yet exist for diagnosis. In our case population, we found that activities suggestive of transient paresthesia, such as looking back urgently as if stung, sitting urgently while on walks, and chewing at the hind limbs or tail, were present in all the dogs, and thus can be used to increase the index of suspicion for TCS. Lack of movement of the CM or DS on dynamic imaging may be an objective measurement that can be used to support a diagnosis of TCS. Half of the dogs in our study also had additional extradural adhesions that required transection in addition to releasing the FT, which has not been reported previously without concurrent NTDs. The results of our study provide more specific clinical features of TCS, and objective imaging findings for diagnosis.

Accepted imaging criteria do not yet exist to diagnose TCS in dogs. In a recent study of 30 dogs with TCS, diagnosis of medically managed dogs was based on normal MRI findings, thus excluding other causes (Supplementary Material S3).^15^ The MRI criteria in affected people include a low-lying CM below L2, with or without a thickened filum terminale (>2 mm).^22^ These criteria cannot be applied to dogs because of substantial variation in the termination level of the CM in dogs and a much thinner, immeasurable FT diameter. Multiple studies have shown that the CM and DS vary in termination levels among individual dogs.^4^^,^^18^^,^^21^ It also is reported that the FT was too thin to be reliably measured on MRI in dogs.^18^ Even when the FTE appeared thickened at surgery, it still was not reported to be abnormal on MRI.^12^ The most common termination site of the CM of dogs in our study was L6, similar to that reported in referenced studies of normal dogs of similar weight.^4^ We found the most common termination site for the DS was L7, whereas previous studies reported the sacrum as the most common DS termination site.^4^^,^^18^ One case report of TCS reported possible caudal dorsal location of the DS as suspicious for TCS,^12^ but in our study only 5/9 dogs had a dorsally located DS in both the flexed and extended views. Therefore, the termination site of the DS and CM, dorsal location of DS, and the size or appearance of the filum are not helpful imaging criteria in diagnosing TCS in dogs.

Our study differed from most prior reports because we used dynamic imaging techniques to diagnose TCS in dogs without explanations for their presenting clinical signs. After surgery and successful outcome, we then documented the extent of craniocaudal movement of the CM and DS in dogs with TCS, when the hips were moved from flexion to extension. This method was first described for diagnosing TCS in a dog in a single case report that evaluated movement of the CM on dynamic MRI. This dog’s heart rate increased during MRI when the hips were flexed, and surgical transection of the FTE led to complete resolution of the dog’s clinical signs at 2-month follow-up.^16^ Only one study evaluated the movement of the CM in 9 healthy dogs using dynamic MRI with flexed and extended pelvic limbs, and found a median CM displacement of 11.6 mm, ranging from 5.5 to 18.8 mm.^20^ Using this same measurement protocol in our dogs with TCS, we confirmed that all the dogs had DS and CM movement of <3 mm. Because the dogs in our study all experienced improvement in clinical signs after surgical untethering, and because the CM moved by at least 5.5 mm in normal dogs on dynamic MRI,^21^ this measurement may help refine objective imaging criteria for diagnosing TCS in dogs*.* Three dogs in our study were diagnosed using dynamic CT, which only allows evaluation of the DS and not the CM. These cases were diagnosed before we had begun objectively measuring movement of the CM and DS, and owners accepted the risks of missing any lesions not visible on CT. Although no studies have quantified the normal degree of movement of the DS in healthy dogs, one of the TCS case reports showed clear movement of the dural sac in a normal dog,^16^ and there also was substantial movement in the control CT referenced (Figure 4). Without quantification in normal dogs, we cannot yet propose a cutoff value for DS movement below which TCS should be suspected, but evaluation of the movement of the DS is likely to be useful with future research.

Our MRI protocol was optimized as we gained experience during our study. We also found it challenging to identify and measure the exact termination levels of the CM and DS, and we could not always identify or measure the FTE, FTI, or both.^21^ Transverse imaging was crucial to maximize the precision in locating the CM and DS, despite some previous studies relying solely on sagittal sequences.^4^^,^^18^^,^^21^ We used the same measurement technique as a previous study that used transverse imaging to help verify the termination of the DS and CM and reported very high interobserver variability.^20^ On initial review of images, transverse sequences helped us to more confidently identify the end of both the CM and DS, especially because the structures taper gradually without an abrupt change in shape that can be identified on sagittal images alone.

One limitation of the technique is that slice thickness will limit measurement accuracy. Our standard T2W transverse protocol acquires 1.5 or 2.5 mm slices, making it impossible to measure distances smaller than that accurately. We improved the accuracy by adopting volumetric sequences, such as the T2W SPACE with a slice thickness of <1 mm, and by using transverse sequences to identify the anatomy. Nevertheless, it was still difficult at times to objectively differentiate the end of the CM from the start of the FT, again because of the tapering nature of this structure and the absence of any abrupt change, which is a limitation of this technique.^18^ Based on our experience and findings, we recommend the following sequences with extension and flexion of the hindlimbs to evaluate dogs with chronic lumbar pain: sagittal T2W, T2W FatSat, and HASTE of the lumbosacral vertebral column, as well as selected transverse T2W and T2W SPACE images over the CM and DS termination sites.

Standard surgical detethering in humans is performed intradurally, with transection of the FTI. In the veterinary literature, transection of only the FTE or both the FTE and FTI is associated with improvement after surgery.^15^ Transection of the FTE is a technically easier procedure and may be less likely to result in complications, such as arachnoiditis or intradural adhesions, and might carry a lower risk of retethering.^22^ Nine of the dogs in our study underwent extradural release without transection of the FTI, and all of them showed improvement in clinical signs with minimal postoperative discomfort, indicating this approach may be a good treatment option in dogs. No studies have compared outcomes for transection of FTE versus FTI in people, but a few small studies have reported good outcomes with only an extradural release.^22^ One cadaver study in humans showed that acute tension on the FTE only caused mild movement of the CM, but chronic tension was not evaluated.^23^ The results of this cadaver study may not apply to dogs for a few reasons. First, tension was applied only briefly, which is not representative of the chronic tension encountered in TCS.^24^ Second, dogs have a much shorter FTI with a longer FTE than do humans. The average length of the FTI in humans is 167 mm, much longer than the FTE, which averages 87 mm,^22^ whereas in dogs, the average FTI length in one study in Cavalier King Charles Spaniels was only 29 mm.^18^ Although FTE length is not well documented in dogs, the FTE is substantially longer than the FTI based on MRI appearance. In addition, signs of TCS in dogs seem also to arise from DS tethering and not just CM tension. The latter hypothesis is supported by the 2 dogs in our series that showed clinical improvement with release of the extradural adhesions alone without transection of either filum, suggesting that DS tension can cause clinical signs of TCS. Half of the dogs in our study also had taut extradural adhesions that we transected during surgery, and which appeared to contribute to tethering. These findings show the importance of releasing these extradural adhesions, meaning that even if an intradural approach is performed, the laminectomy should be opened sufficiently caudally to identify extradural adhesions*.* Many of these extradural adhesions were very small and difficult to visualize, and thus careful evaluation of the area around the dural sac should be performed during surgery. Based on our results, isolated FTE transection can lead to clinical improvement in dogs with TCS. Nevertheless, the precise cause of tethering likely varies from dog to dog. With further experience, we hope to tailor the surgical procedure to each dog.

All 12 dogs in our series showed substantial improvement at short-term follow-up, supporting a diagnosis of TCS. At long-term follow-up, 11 dogs showed improvement, whereas 1 dog was lost to follow-up (Supplementary Material S4). These outcomes are similar to those reported in humans, who also commonly report substantial improvement without complete resolution of clinical signs. One review in affected adults reported that pain was the clinical sign most responsive to surgery, with 82% of people having improvement postoperatively, whereas only 62% of motor deficits and 45% of bladder signs showed improvement.^11^ In our case series, we also found that pain was very responsive to surgery, with 100% of the dogs having improvement in back pain and 89% of the dogs showing improvement in reluctance to jump or climb stairs. Episodes of presumed transient paresthesia also responded well to surgery, with 100% of these signs showing improvement or resolution after surgery. Gait was the least responsive to surgery, with only 73% of signs (stiff gait, bunny-hopping, and lameness) improving with surgery, similar to the persistent motor deficits and neurologic signs reported in people. All the owners subjectively reported substantially improved quality of life after surgery at long-term follow-up, despite many reporting only partial improvement of some signs.

Our study had some limitations. Our primary goal was to develop an objective MRI measurement protocol to help diagnose TCS, but the lack of control animals was limiting. We had to rely on the sparse literature showing translocation of the CM in normal dogs, with no corresponding data available on DS movement. Additional studies evaluating CM and DS movement in normal dogs of different breeds and sizes using dynamic MRI could help confirm our proposed cutoff values to diagnose TCS. The surgical procedure chosen was also variable, evolving as we gained more experience in subsequent cases. Thus, we cannot compare the different approaches for detethering, particularly in terms of whether or not it is helpful to section the FTI. Our study was also retrospective, with variability in the nature and duration of follow-up. Longer observation periods also will be needed to evaluate the rates of complications such as retethering, a common complication in human patients.

In summary, TCS is likely to be an underdiagnosed condition in dogs because of the wide anatomical variations among dogs, a general lack of awareness of presenting clinical signs, and lack of consensus on imaging diagnosis. All the dogs in our study had DS and CM movement of <3 mm. Because normal CM movement was at least 5.5 mm in normal dogs on dynamic MRI, this measurement may lead to objective criteria to diagnose TCS. Dogs with chronic lumbar pain, poor exercise tolerance, and transient paresthesias should undergo dynamic MRI, including transverse imaging through the CM and DS with the hips in flexion and extension. If <3 mm of craniocaudal movement of the CM and DS is found with no other lesions to explain the signs, a diagnosis of TCS should be considered. Surgical release of tethering structures may lead to improved quality of life.

## Supplementary Material

aalaf031_Supplemental_Files

## Off-label antimicrobial declaration

Authors declare no off-label use of antimicrobials.

## Institutional animal care and use committee or other approval declaration

The authors declare that no institutional animal care and use committee or other approval was needed.

## Abbreviations

CM: conus medullaris
CT: computed tomography
DS: dural sac
FT: filum terminale
FTE: filum terminale externum
FTI: filum terminale internum
HASTE: half-Fourier acquisition single-short turbo spin-echo
MRI: magnetic resonance imaging
NTD: neural tube defects
SPACE: sampling perfection with application-optimized contrast using different flip angle evolution
TCS: tethered cord syndrome
