# Supplementary material for: Diagnosis, surgery, and outcome of tethered cord syndrome in 12 dogs
Source: J Vet Intern Med. 2026 Jan 21;40(1):aalaf031. doi: 10.1093/jvimsj/aalaf031 (PMC12881950; doi:10.1093/jvimsj/aalaf031)
Supplement: aalaf031_Supplemental_Files [file aalaf031_supplemental_files.zip › S1_Survey_long_term_outcome_aalaf031.docx]

- What is your dog's name?
- These are the most common clinical signs we have compiled from dogs with tethered cord syndrome. Please answer if you felt these signs improved or resolved with surgery. Choose N/A if your dog did not have these signs before surgery.

|  | N/A (Not present before surgery) | No change or worse after surgery. | Partial resolution of clinical sign | Complete resolution of sign after surgery |
| --- | --- | --- | --- | --- |
| Back pain |  |  |  |  |
| Signs that worsen with exercise |  |  |  |  |
| Stiff gait |  |  |  |  |
| Looking back urgently/as if stung |  |  |  |  |
| Exercise intolerance |  |  |  |  |
| Chewing at feet/hind end/tail |  |  |  |  |
| Bunny hopping gait at times |  |  |  |  |
| Reluctance to jump or do stairs |  |  |  |  |
| Sitting suddenly/urgently on walks |  |  |  |  |
| Lameness |  |  |  |  |
| Anxiety/behavior disorders |  |  |  |  |
| Incontinence |  |  |  |  |

- Do you feel like their quality of life is improved compared to before surgery?

Yes No Equivocal

- What medications are they currently on?
- Other comments about your dogs outcome following tethered cord surgery.
