# Supplementary material for: Diagnosis, surgery, and outcome of tethered cord syndrome in 12 dogs
Source: J Vet Intern Med. 2026 Jan 21;40(1):aalaf031. doi: 10.1093/jvimsj/aalaf031 (PMC12881950; doi:10.1093/jvimsj/aalaf031)
Supplement: aalaf031_Supplemental_Files [file aalaf031_supplemental_files.zip › S2_Table_of_dogs_aalaf031.docx]

Supplementary material 2: Signalment, owner observation and neurological exam of 12 dogs who underwent TCS surgery.

|  | Age at presentation (months) | Signalment | Gender | Weight (kg) | Onset of Signs (months) | Duration of Signs (months) | Incontinence | Comorbidities | Other notes from owner | Neurological exam abnormalities | Orthopedic Exams |
| --- | --- | --- | --- | --- | --- | --- | --- | --- | --- | --- | --- |
| 1 | 24 | Welsh Corgi | MI | 13 | 18 | 6 |  | None | Can’t stand on one leg to pee, painful to touch n L Hip. Tires and lays down on walks. Looks back suddenly as if stung | Moderate paraparesis, mild ataxia; absent placing in both pelvic limbs; resistant to hip extension | Neurologist: Pain on hip extension |
| 2 | 62 | Suberian Husky X | MN | 29.5 | 24 | 38 |  | None | Scuffing of pelvic limbs occasionally, no tail wagging, won’t roll on back, severe anxiety and aggression, uncomfortable pooping and poops while standing. Sits suddenly on walks, chews at back legs, tail, feet, bunny hopping, sensitive to touch in hind end | Stiff gait; pain with hip extension and lumbar palpation with lordosis | Neurologist: Pain on hip extension |
| 3 | 51 | Labrador Retriever | FS | 22.15 | 32 | 19 |  | Gastroenteritis | Right leg muscle side appeared smaller than left. Sits suddenly on walks, chews at back end, sensitive to touch on back end, looks back as if stung, bunny hops | Stiff gait in LPL; weak withdrawal and lumbar pain on lordosis | Neurologist: Normal exam |
| 4 | 51 | Australian Shepherd | MN | 23.6 | 38 | 13 |  | None | Knuckling occasionally; muscle spasms especially when defecating; shifting weight right to left; progressively stiff hind limbs, sat suddenly on a awalk | Stiff gait, inability to fully extend or flex hips; possible reaction to cervical and lumbar palpation | Surgeon: Resistant to hip extension bilaterally, mild right hock effusion, no overt abnormalities on hip/stifle rads |
| 5 | 39 | Australian Shepherd | MN | 18.8 | 3 | 36 |  | None | Anxiety severe; Short stilted gait of thoracic limbs and pelvic limbs eventually, licks at feet | Stiff pelvic limb gait, delayed placing in rear | Surgeon: Resistant/pain on hip extension bilaterally, no other abnormalities |
| 6 | 145 | Terrier | MN | 9.55 | 126 | 19 |  | None | Excessive discomfort at night, unable to sit in frog legged position; anxiety, chews at feet, looks back as if stung, stiff gait | Stiff gait; decreased withdrawals bilaterally; lumbar pain, pain on hip extension | Neurologist: Pain on hip extension |
| 7 | 52 | Australian Shepherd | MN | 27.45 | 3 | 49 |  | None | Chewing excessively; worse with walks, sits urgently on walks, chews at feet excessively, looks back as if stung, bunny hops | Only lumbar pain on lordosis | Neurologist/rehab: Iliopsoas pain, otherwise normal; |
| 8 | 68 | Formosan Mountain Dog X | FS | 13 | 30 | 38 | yes - dribbled or emptied her bladder when lying down or sleeping | None | Sits urgently on walks, chews at feet, anus, tail, looks back as if stung, bunny hops, intermittent lameness | Pain on lordosis, looks back when touched in hind end | Surgeon: Iliopsoas pain, otherwise normal |
| 9 | 89 | Golden Retriever | MN | 35 | 47 | 42 |  | None | Intermittent left forelimb lameness, hesitant to do lumbar stretch, looks back as if stung, sits urgently on walks, bunny hops | Pain on lordosis and tail jack; no deficits | Neurologist: Pain on hip extension |
| 10 | 53 | Husky X | FS | 22.7 | 14 | 39 | yes - dribbled transiently while walking and immediately after producing a normal stream of urine | Mild bilateral supraspinatus tindinopathy (historical) | Back spasm when being pet, kicking out legs while walking, occasionally chews at tail or feet, looks back sometimes as if stung | Stiff pelvic limb gait, pain on lordosis | Neurologist: No abnormalities; rehab specialist: pain on hip pextension; historical surgery consult revealed mild forelimb lameness that was resolved |
| 11 | 22 | German Shepherd | F | 29.7 | 2 | 20 |  | none | Anxiety (light sensitive, touch sensitive); suddenly yelps and looks scared, chews at feet, looks back as if stung, bunny hops, intermittent lameness, stiff gait | Pain on lordosis, no deficits | Neurologist/rehab: Normal exam |
| 12 | 57 | Boston Terrier | MN | 10.7 | 1/23 | 36 | yes - fecal and urinary incontinence on days that he became more painful | Hydrocephalus, supracollicular fluid accumulation, block vertebrae at C2-3 and C4-5 | Long term history of neck pain (kyphosis, unable to move neck); new onset of reactivity around hind end (screams at gentle touch), knuckled a couple of times in pelvic limbs, kicking legs out when walking, sits urgently on walks, chews at feet. | Pain on lumbar palpation | Neurologist: Normal exam |
