# Supplementary material for: Diagnosis, surgery, and outcome of tethered cord syndrome in 12 dogs
Source: J Vet Intern Med. 2026 Jan 21;40(1):aalaf031. doi: 10.1093/jvimsj/aalaf031 (PMC12881950; doi:10.1093/jvimsj/aalaf031)
Supplement: aalaf031_Supplemental_Files [file aalaf031_supplemental_files.zip › S3_and_S4_aalaf031.docx]

Supplemental information 3: Recent publication of case series of 30 dogs with TCS.

A study describing 30 dogs with TCS was published after initial submission of our manuscript. These results were not initially available for comparison at the time of design and writing our series. The authors of that study reported very similar presenting clinical signs to those found in our cases, with the most common finding including pain, gait abnormalities, impaired physical activity and behavioral changes.^16^ The outcomes following surgery were also similar to ours, with all dogs showing improvement following transection of the FTE, and/or FTI. Nevertheless, at present the most challenging aspect of TCS in dogs is making a definitive diagnosis. In contrast to our study where all dogs underwent dynamic imaging and surgery, the multicenter, retrospective nature of the 30 dogs meant that only 11 had their diagnosis confirmed at surgery, and only 20 had dynamic imaging. In addition, the results of imaging were reported simply as a subjective lack of cranio-caudal movement of the CM rather than a quantitative measurement between flexion and extension. In our study we elected to restrict inclusion to dogs that had both surgical confirmation and we provided quantitative measurements of CM and DS movement. By keeping our criteria restrictive, the aim of our study is to develop a framework for more objective means to confirm the diagnosis in future cases.

Supplementary information 4: Discussion of discrepancies between outcomes when reported via survey or in person.

It was interesting to note there were some discrepancies between owner’s answers on our survey and those provided in person. For example at an in person recheck one owner said their dog had been pain free for months, but that same week filled out the survey and noted only partial recovery of back pain. Studies on occult TCS in humans found similar contradictory findings when evaluating outcomes of surgery with patient reported symptoms versus clinical assessment.^25,26^ There are a number of variables that could contribute to this discrepancy, such as the wording of the questions used in both survey and in person, and the owner’s interpretation of the question. During the study design we felt that a standardized survey presented to each patient would provide a more objective measurement outcome than an informal follow up. However, our findings illustrate the importance of the survey design and challenges of comparing outcomes collected in different formats. For our ongoing prospective studies we plan to use a validated pain questionnaire such as the Canine Brief Pain inventory, and to include ‘frequency’ and ‘intensity’ into the questions asked of the owner.
